# The Relationship Between the Home Environment and Falls for Older Adults With Visual Impairments

**DOI:** 10.1093/geroni/igaa057.2783

**Published:** 2020-12-16

**Authors:** Bonnielin Swenor, Aleksandra Mihailovic, Pradeep Ramulu

**Affiliations:** 1 Johns Hopkins University, Baltimore, Maryland, United States; 2 Johns Hopkins University/Wilmer Eye Institute, Baltimore, Maryland, United States; 3 Johns Hopkins School of Medicine, Baltimore, Maryland, United States

## Abstract

The home environment and features of the home have been identified as important risk factors for falls, and may pose particular risk for older adults with visual impairments given difficulty with hazard perception. We used data from 245 participants in the Falls in Glaucoma Study [mean age: 71 years, mean follow-up: 31 months] with homes graded using our previously validated Home Environment Assessment for the Visually Impaired (HEAVI), which quantifies the number of in-home fall-related hazards and found that neither the number of hazards nor the percentage of hazardous items were associated falls/year. However, each 10-fold increase in lighting was associated with a 35% lower rate of falls/year (RR=0.65, 95%CI=0.46 to 0.92) and there was a 50% reduction in falls/year when lighting was at or above 30 footcandles (minimum lighting level recommended by the Engineering Society of North America) compared to lighting <30 footcandles (RR=0.50, 95%CI=0.26 to 0.96).

